# 1733. Effectiveness of Influenza Vaccination Against Influenza-Associated Emergency Department (ED) Visits and Hospitalizations Among Children With and Without Underlying Medical Conditions, New Vaccine Surveillance Network (NVSN), 2015-2016 through 2019-2020 Influenza Seasons

**DOI:** 10.1093/ofid/ofad500.1564

**Published:** 2023-11-27

**Authors:** Emma K Noble, Haya Hayek, Laura S Stewart, Leila C Sahni, Julie A Boom, Marian G Michaels, John V Williams, Janet A Englund, Eileen J Klein, Mary A Staat, Elizabeth P Schlaudecker, Rangaraj Selvarangan, Jennifer E Schuster, Geoffrey A Weinberg, Peter G Szilagyi, Benjamin R Clopper, Heidi L Moline, Natasha B Halasa, Samantha M Olson

**Affiliations:** Centers for Disease Control and Prevention, Atlanta, Georgia; Vanderbilt University Medical Center, Nashville, Tennessee; Vanderbilt University Medical Center, Nashville, Tennessee; Baylor College of Medicine and Texas Children’s Hospital, Houston, Texas; Texas Children’s Hospital, Houston, Texas; UPMC Children's Hospital of Pittsburgh, Pittsburgh, Pennsylvania; University of Pittsburgh, Pittsburgh, Pennsylvania; Seattle Children’s Hospital, Seattle, Washington; University of Washington School of Medicine, Seattle, Washington; Cincinnati Children’s Hospital Medical Center, Cincinnati, Ohio; Cincinnati Children's Hospital Medical Center, Cincinnati, Ohio; Children’s Mercy Kansas City, Kansas City, Missouri; Children’s Mercy Kansas City, Kansas City, Missouri; University of Rochester School of Medicine & Dentistry, Rochester, NY; UCLA School of Medicine, Agoura Hills, California; US Centers for Disease Control & Prevention, Buffalo, New York; Centers for Disease Control and Prevention, Atlanta, Georgia; Vanderbilt University Medical Center, Nashville, Tennessee; Centers for Disease Control and Prevention, Atlanta, Georgia

## Abstract

**Background:**

Children with underlying medical conditions are at higher risk for developing severe influenza illness. Annual influenza vaccination is recommended for all children ≥ 6 months and can protect against severe influenza. Limited studies have examined if vaccine effectiveness (VE) against influenza illness differs in children with underlying conditions compared to those without.

**Methods:**

We used a test-negative design to assess VE against laboratory-confirmed influenza-associated ED visits or hospitalizations in children 6 months-17 years of age with and without underlying conditions. Children were enrolled at 7 medical centers within NVSN, a prospective respiratory viral surveillance platform in 7 states, during each influenza season from 2015-2016 through 2019-2020. Influenza vaccination was assessed using documentation from state registries or providers. Underlying conditions were abstracted from medical records. We estimated VE by comparing the odds of vaccination ≥ 14 days prior to symptom onset in case patients with influenza compared to test-negative control patients with non-influenza respiratory illness. VE was adjusted for age, study site, and calendar time.

**Results:**

A total of 15,739 children were included (2,800 cases and 12,939 controls). Among cases, 48% had an underlying condition, and about half (51%) of those conditions were respiratory (Table). Cases with underlying conditions were more likely to be hospitalized (53%) compared to cases without (26%). Among patients with underlying conditions, 38% of cases and 56% of controls were vaccinated (Figure). Overall (ED or hospitalization) VE was 48% (95% CI: 41%, 54%) among children with underlying conditions and 57% (95% CI: 50%, 62%) among children without. VE against hospitalization was 46% (95% CI: 36%, 54%) among children with underlying medical conditions and 59% (95% CI: 48%, 68%) among children without.

**Figure:**
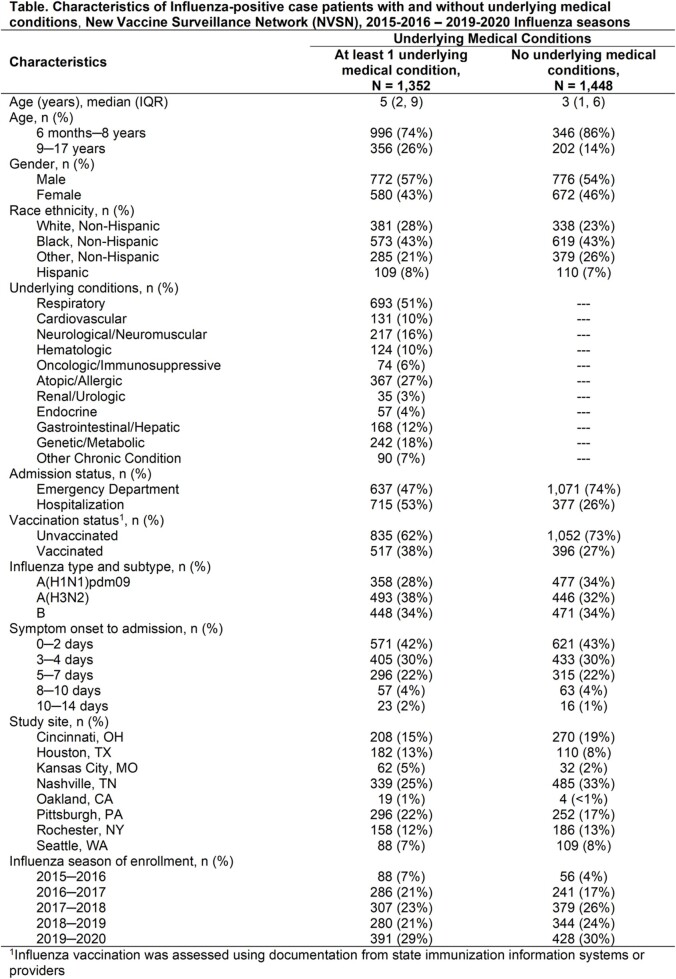

**Figure:**
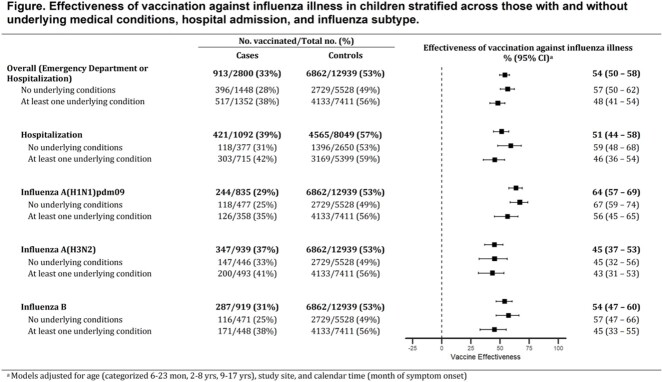

**Conclusion:**

Vaccination reduced the odds of influenza illness in children with and without underlying conditions. VE was similar in children regardless of presence of conditions. Vaccination was sub-optimal in children with and without underlying conditions and continued efforts are needed to improve vaccination uptake in all children ≥ 6 months.

**Disclosures:**

**Marian G. Michaels, MD, MPH**, Merck: Grant/Research Support|Viracor: Grant/Research Support **John V. Williams, MD**, Merck: Grant/Research Support|Quidel: Board Member **Janet A. Englund, MD**, Ark Biopharma: Advisor/Consultant|AstraZeneca: Advisor/Consultant|AstraZeneca: Grant/Research Support|GlaxoSmithKline: Grant/Research Support|Meissa Vaccines: Advisor/Consultant|Merck: Grant/Research Support|Moderna: Advisor/Consultant|Moderna: Grant/Research Support|Pfizer: Advisor/Consultant|Pfizer: Grant/Research Support|Sanofi Pasteur: Advisor/Consultant **Mary A. Staat, MD, MPH**, CDC: Grant/Research Support|Cepheid: Grant/Research Support|Merck: Grant/Research Support|NIH: Grant/Research Support|Pfizer: Grant/Research Support|Up-To-Date: Honoraria **Elizabeth P. Schlaudecker, MD, MPH**, Pfizer: Grant/Research Support|Sanofi Pasteur: Advisor/Consultant **Rangaraj Selvarangan, BVSc, PhD, D(ABMM), FIDSA, FAAM**, Abbott: Honoraria|Altona Diagnostics: Grant/Research Support|Baebies Inc: Advisor/Consultant|BioMerieux: Advisor/Consultant|BioMerieux: Grant/Research Support|Bio-Rad: Grant/Research Support|Cepheid: Grant/Research Support|GSK: Advisor/Consultant|Hologic: Grant/Research Support|Lab Simply: Advisor/Consultant|Luminex: Grant/Research Support **Geoffrey A. Weinberg, MD**, Merck & Co: Honoraria **Natasha B. Halasa, MD, MPH**, Merck: Grant/Research Support|Quidell: Grant/Research Support|Quidell: donation of kits|Sanofi: Grant/Research Support|Sanofi: vaccine support

